# Multidisciplinary Approach to Interstitial Lung Diseases: Nothing Is Better than All of Us Together

**DOI:** 10.3390/diagnostics10070488

**Published:** 2020-07-17

**Authors:** Carlo Vancheri, Antonio Basile

**Affiliations:** 1 Department of Clinical and Experimental Medicine, University of Catania, Regional Referral Center for Rare Lung Diseases, University Hospital “Policlinico–Vittorio Emanuele”, via S. Sofia 78, 95123 Catania, Italy; 2 Department of Medical Surgical Sciences and Advanced Technologies, Radiology Unit I, University Hospital “Policlinico–Vittorio Emanuele”, 95123 Catania, Italy; basile.antonello73@gmail.com

## Abstract

Interstitial Lung Diseases (ILDs) are a large family of disorders characterized by inflammation and/or fibrosis of areas of the lung dedicated to gas exchange. In this Special Issue entitled “Clinical and Radiological Features of Interstitial Lung Diseases”, we collected a series of contributions in which a multidisciplinary approach was crucial for the correct diagnostic assessment of ILD. Sharing knowledge between different specialties can significantly improve diagnostic approaches and the management of ILD patients.

Interstitial Lung Diseases (ILDs) are a large family of disorders characterized by inflammation and/or fibrosis of areas of the lung dedicated to gas exchange. The impairment of lung parenchyma causes the occurrence of symptoms, and in some ILDs, such as idiopathic pulmonary fibrosis (IPF), it may lead to respiratory failure and death. Some ILDs, including IPF and sarcoidosis, are idiopathic, others such as hypersensitivity pneumonia and drug-induced ILDs recognize specific etiologic agents, some others are instead associated with systemic diseases, as in the case of connective tissue diseases (CTD-ILDs) [1]. ILDs represent about 20% of lung diseases routinely seen by pulmonologists during their daily activity, and their number, due to the improvement of the diagnostic procedures, is increasing [2]. It is also plausible that exposures to known risk factors such as smoking, but also new and unknown environmental and professional agents or drugs may be responsible for this alarming trend. All this may explain the increasing scientific attention to ILDs by researchers and clinicians, attracted by the wide array of “intriguing” basic and clinical issues associated with pathogenesis, diagnosis, follow-up, and treatment of this group of diseases. In particular, the large number of clinical, functional, and radiological overlaps among ILDs makes their diagnosis extremely difficult and challenging. For these reasons, a multidisciplinary approach to the diagnosis of ILDs is strongly recommended, representing the best way to improve the diagnostic accuracy of these diseases [3]. In this Special Issue, dedicated to the “Clinical and Radiological Features of Interstitial Lung Diseases”, the two editors present a series of articles where pulmonologists, radiologists, rheumatologists, and other experts, put their knowledge together with the unique aim of underlining specific clinical and radiological aspects of ILDs that might be useful to the diagnostic approach to ILDs for a larger audience of clinicians. Some of the articles in this Special Issue are opportunely dedicated to High-Resolution Computed Tomography (HRCT), one of the most important diagnostic tools in ILDs. In most ILDs, symptoms are non-specific, making the differential diagnosis particularly difficult. Only in some cases is the radiological pattern typical for a specific disease, whereas in many others, the interpretation of the radiological picture is more difficult and needs to be reviewed by an expert lung radiologist. In any case, the interpretations based on qualitative evaluations of HRCT are limited by a visual analysis, which is time-consuming, and above all, subjective. Stefano et al. show in this issue that computer-based analysis might be a useful tool for the radiological assessment of patients with IPF. In particular, they found a specific parameter measuring the percentage of normally attenuated lungs that, together with qualitative HRCT assessment and pulmonary function test, increases the accuracy of the diagnostic process in IPF [4]. The whole matter is further complicated by the pleiomorphisms of radiological presentations in different ILDs, but also in the same disease at different stages. Tiralongo et al. describe the evolution of morphological patterns at HRCT in cryptogenic organizing pneumonia in response to treatment with steroids [5]. Aquilina et al. have instead focused their attention on cystic lung diseases, one of the most typical radiological presentations of ILDs [6]. Their study aims to offer a practical guide to radiologists and pulmonologists for an easier interpretation of these particular lesions. To do this, the different cystic lesions were effectively and nicely described through free-hand drawings that are related to HRCT images, helping to recognize the correct differential diagnosis between similar conditions. Sambataro et al. observed that ground-glass opacities at HRCT correlate with disease activity in Systemic Sclerosis (SSc), whereas the fibrosis grade correlated with disease duration and pulmonary artery pressure [7]. Besides, they observed that the quantification of pulmonary involvement using the Wells score could be a useful tool for assessing the appropriate treatment in SSc patients. Remaining in the field of SSc and autoimmune diseases, Sambataro et al. also present some interesting data on nailfold videocapillaroscopy, described in this manuscript as a useful tool for the recognition of SSc and idiopathic inflammatory myopathies in patients with ILDs [8]. Furthermore, in a different article of this Special Issue, the role of the rheumatologist within the multidisciplinary team is underlined, and how a set of first-line clinical, laboratory, and instrumental tests might increase the frequency of diagnosis of autoimmune diseases among patients with ILD [9]. In another interesting paper, Galioto et al. described the most peculiar clinical and radiological features of complications in IPF [10]. The last two articles, distinct from all the others, are dedicated to the possible relationship between the radiological patterns of sarcoidosis and its clinical outcome, and the last article is an interesting description of HRCT patterns in drug-induced ILDs [11,12].The diagnosis of drug-induced-ILDs, as a consequence of the introduction of new drug therapies, is constantly increasing. Unfortunately, the morphological HRCT patterns are not specific for a given drug. Therefore the diagnosis should be based on clinical-anamnestic data, radiological, and laboratory features, confirming once again the need for a multidisciplinary team approach for the diagnosis of ILD.

All articles of this Special Issue are comprehensive and innovative in their approaches, addressing practical and still controversial or unresolved clinical problems related to the diagnosis, follow-up, and prognosis of ILDs. The two editors, Stefano Palmucci and Sebastiano Torrisi, both have the merit to involve and coordinate in this Special Issue, a number of young clinicians and experts. Looking at the list of authors who contributed to the different articles, it is nice to note the variety of their specialties: Biologists, physicists, immunologists, rheumatologists, radiologists, internists, computer scientists, and pulmonologists have all contributed to the success of this issue dedicated to ILDs. The participation and commitment of young experts to this group of diseases, coming from very different scientific settings, confirm the relevance and the broad clinical interest for this important chapter of the respiratory diseases.

## Abbreviations

CTD	Connective Tissue Disease
HRCT	High-Resolution Computed Tomography
ILD	Interstitial Lung Diseases
IPF	Idiopathic Pulmonary Fibrosis
MDT	Multidisciplinary Team
SSc	Systemic Sclerosis.
